# Magnitude and correlates of moderate to severe anemia among adult HIV patients receiving first line HAART in Northwestern Tanzania: a cross sectional clinic based study

**DOI:** 10.11604/pamj.2016.23.26.8268

**Published:** 2016-02-04

**Authors:** Daniel Wilfred Gunda, Semvua Bukheti Kilonzo, Bonaventura Cornel Mpondo

**Affiliations:** 1 Department of Medicine, Weill Bugando School of Medicine, Mwanza, Tanzania; 2 Department of Medicine, College of Health Sciences, University of Dodoma, Dodoma, Tanzania

**Keywords:** HIV/AIDS, anemia, risk factors, antiretroviral therapy, Tanzania

## Abstract

**Introduction:**

Moderate to severe anemia is an important clinical problem in HIV patients on Highly Active Antiretroviral Therapy. The rate of progression and mortality in this sub group of patients is high compared to non anemic patients. In sub Saharan Africa with scale up of Anti retroviral therapy, the magnitude of this problem is not known especially in Tanzania. This study aimed at determining the magnitude and correlates of moderate to severe anemia in HIV patients receiving first line ART in northwestern Tanzania.

**Methods:**

This was a cross sectional clinic based study, involving adult HIV patients on first line Highly Active Antiretroviral Therapy at Bugando Medical Centre Care and Treatment Center. The patients’ data were analyzed using STATA version 11 to determine the prevalence of moderate to severe anemia and risk factors that could predict occurrence of anemia.

**Results:**

In this study 346 patients on Highly Active Anti-Retroviral Therapy were enrolled, of whom 100(40.46%) had moderate to severe anemia. The odds of being anemic were strongly predicted by Zidovudine based regime, low baseline CD4 count (< 200 cells/μl) and HIV stage 3&4 at enrollment. Most of the anemic patients had mean corpuscular volume of >100fl.

**Conclusion:**

The prevalence of moderate to severe anemia is significantly high in this cohort of HIV-infected patients on first line Anti Retroviral Therapy and it is strongly predicted by Zidovudine based regime, low baseline CD4 and HIV stage 3 and 4. On clinical grounds this suggests that patients who are initiated on Zidovudine based regimen and those in advanced HIV at enrollment should have regular haemoglobin follow up to identify anemia at its earliest stage to improve the clinical outcome of these patients.

## Introduction

Human immune deficiency virus/ acquired immune deficiency syndrome (HIV/AIDS) is still a global problem, which despite the use of highly active antiretroviral therapy (HAART) to reduce AIDS related events, it still has high morbidity and mortality mediated through non immunological complications of HIV/AIDS. Anemia is the most common hematological complication in HIV patients [1–3]. The prevalence of anemia in HIV patients is significantly high. Among ART naïve HIV-infected patients some study settings record a prevalence of more than 70%( 18-95%). For example Daka et al. reported a prevalence of anemia in 86.5% of HAART naïve patients in Ethiopia [4]. Frontiera and colleagues studying the peripheral blood of HIV patients for abnormalities, anemia was noted in 95% of the patients samples [2]. While Johannessen reported a prevalence of 77.4% among adult HAART naïve patients in rural Tanzania [5]. Initiation of HAART has usually been regarded as a standard management of HIV/AIDS patients. In addition to restoration of immunological function of the body to fight against opportunistic infections, HAART have also been shown to improve HIV related hematological complications especially anemia [5–7]. But even with HAART still a considerable proportion of patients on HAART have anemia with serious clinical implications. One study in Ethiopia demonstrated only a 6% reduction of anemia from a pre HAART prevalence of 86.5% to 80.5% after ART [4], with significantly high prevalence of moderate and severe anemia (62.7%). Omoregie demonstrated a prevalence of anemia in 69.17% and 51.15% of HAART naïve and HAART experienced patients respectively [8]. While Johannessen et al, recorded a prevalence of 77.4% of anemia in HAART naïve patients, where 38.2% still remained anemic after 12 months of HAART [5].

Clinical implications of anemia include decreased survival time and HIV disease progression. In euroSIDA study, the survival rate at 12months in HIV patients with mild anemia, (Hb<12g/dl in females and Hb <13g/dl in men), was significantly shorter than in non anemic counter parts (84.1% vs. >96.9%) and it was even much shorter among severely anemic patients (HB<8g/dl) [9]. Sullivan and colleagues in their study involving more than 32000 patients found that the survival rate was significantly reduced in HIV anemic patients with moderate to severe anemia (Hb<10g/dl) with a relative risk of death of 148% [1]. From available studies it is important to note that these implications are more serious in patients with moderate and severe anemia, but also it has been shown that HIV/AIDS patients with moderate to severe anemia suffer a rapid HIV progression to AIDS. In euroSIDS study studying the effect of anemia in HIV progression to AIDS, patients with severe anemia had a high relative hazard of disease progression as compared to those with mild anemia (7.1 vs. 2.2) [10]. Correction of anemia has been shown to improve symptoms and quality of life. The available literature recommends use of epoetin until normal Hb is restored especially in a situation where correctable cause is not apparent [11, 12]. However most of these measures may still be expensive and not readily available for routine practical use especially in Tanzania and sub Saharan Africa at large where HIV burden is highest and causes of anemia are multi factorial. These areas may probably benefit more from early identification through routine screening of potential patients and make timely clinical follow up before severe state of anemia is reached. In the background of this information though there has been a significant scaling up of HAART and CTC activities, however the literature on the magnitude of moderate to severe anemia in patients on HAART is still scarce especially in Tanzania. The aim of this study was therefore to determine the proportion and correlates of moderate to severe anemia in patients receiving first line HAART in northwestern part of Tanzania.

## Methods

### Study design and setting

This was a cross sectional cohort clinic based study done at Bugando medical centre (BMC) between May 2011 and April 2012. BMC is a tertiary level and teaching hospital for the North Western part of Tanzania. It has a capacity of 1000 beds, and serving around 16 million people. Bugando is also a HIV referral center running its CTC activities as core part of outpatient activities. The center started a way back 2004, and it currently serves a total of more than 10,000 patients, whereby about 5000 of them are active on Antiretroviral therapy.

### Sample size and sampling procedure

A minimum sample size of 246 was calculated assuming a prevalence of moderate to severe anemia to be 20% with a range of 10-80.5% in available studies [4, 13] and patients were serially enrolled after consent until the sample size was reached.

### Study population

This study included all adult HIV patients on first line ART ageing 18 years and above who attended a care and treatment center (CTC) at Bugando in Mwanza, Tanzania between May 2011 and April 2012. Patients who were younger than 18years or on second line ART, and those who were not yet on ART, pregnant women, seriously ill patients and those who did not consent for the study were excluded.

### Data collection and statistical analysis

After consent the information of interest was recorded in a special tool including the demographic data, year of HIV diagnosis, year of start of ART, the ART regimen, height and body weight, a record of most recent Hb and red blood cell (RBC) indices, baseline CD4 and current CD4. Data were computerized using Epi data version 3.1 and STATA version 11 (Stata Corp LP, college station, TX) was used for data analysis. The effect of different risk factors on the odds of having moderate to severe anemia was investigated. Odds ratio with 95% confidence interval was used to quantify the strength of association between moderate to severe anemia and its potential predictors. In all of our analyses factors were considered significantly associated with the outcome variable when the p value was<0.05.

### Definition of variables

In this study the first line HAART was referred to as a combination of 2 Nucleoside reverse transcriptase and one non nucleoside reverse transcriptase inhibitor or a protease inhibitor with or without a pharmacological booster. And anemia was defined based on WHO hematological reference values for adults [14]. Accordingly, anemia was defined as Haemoglobin (Hb) concentration less than 13 g/dl for adult males and less than 12g/dl for adult females and by category of severity mild, moderate and severe anemia were defined as Hb concentration of 10-12/13g/dl, 8-10g/dl and <8g/dl respectively, and those patients who had Hb level <10g/dl were referred as having moderate to severe anemia for both sexes.

### Ethical consideration

All study participants were asked for consent, only those patients who consented were included in this study. Patients who were found to have severe anemia and on Zidovudine (AZT) regimen were changed to non AZT based regimen and investigations for probable cause of anemia were done including stool for worms. The study was approved by Bugando Medical Centre/Catholic University of Health and Allied Sciences (BMC/CUHAS) joint research committee.

## Results

### Characteristics of study participants

A total of 3048 patients were screened for study eligibility, with an average of 40 patients seen every day. Of these; 2702 patients were excluded due to different reasons including being younger than 18 years (686 patients), not yet on ART (1780 patients), seriously sick (80 patients), on second line ART (166 patients), and refusal to consent for the study (40 patients). In this study a total of 346 patients were included of whom most patients were females, 214(61.85%) with a median age of 41.2(19-66) and median BMI of 22.3(14.8-37.9). More than 50% of study participants were in WHO clinical stage 3&4 with a median time on ART of 44.5(3-69) months and a mean baseline CD4 163.8(5-380) cells/μl. The most common ART combination was Tenofovir (TDF) + Emtricitabine (FTC) + Efavirenz (EFV) (Table 1). Most patients with moderate to severe anemia were also found to have macrocytosis (Mean corpuscular volume >100fl) as compared to those with mild anemia or non anemic counter parts (37.86% vs. 11.17%, p=0.001).

**Table 1:** Basic socio-demographic and clinical characteristics of 346 HIV positive patients on first line HAART

Factors	Median (IQR) or number (%)
Age	41.2(19-66)
Gender	
Male	132(38.15)
Female	214(61.85)
BMI (Kg/M^2^)	22.3(14.8-37.9)
WHO stage	
1&2	197(56.94)
3&4	149(43.06)
Time on ART (mo)	44.5(3-69)
Baseline CD4 (cell/μl)	163.8(5-380)
Enrolment CD4 (cell/μl)	239.4(8-783)
HB levels	
Non anemic	101(29.19)
Mild anemia	105(30.35)
Severe anemia	049(14.16)
ART combinations	
TDF + FTC + EFV	138(39.88)
AZT + 3TC + EFV	091(26.30)
AZT + 3TC + NVP	060(17.34)
d4T + 3TC + NVP	048(13.87)
TDF + FTC + NVP	004(01.16)
TDF + FTC + LPV/r	003(00.87)
AZT + 3TC + LPV/r	002(00.58)
AZT base regimen	153(44.22)
TDF base regimen	145(41.91)
D4T base regimen	048(13.87)

* WHO World health Organization, CD4 Cluster of differentiation number 4, HB hemoglobin, Mo months

### Moderate to severe anaemia and predictors

In general 205(70.71%) of the study participants were anemic and 100(40.46%) had moderate to severe anemia, which was mostly macrocytic in type (37.86 vs. 11.17%, p=0.001) and was independently associated with AZT based regimen (OR=3.3, p=0.005), advanced clinical stage (OR=5.3, p<0.001) and low baseline CD4 count (OR=2.27, p=0.003). The distribution of other factors had no significant statistical difference (Table 2)

**Table 2:** Univariate and multivariate analysis of factors associated with moderate to severe anemia in 138 Adult HIV patients receiving first line HAART

Predictive factor	Hemoglobin levels		Unadjusted		Adjusted	
	HB<10g/dl (N=138)	HB>10g/dl(n=208)	OR(95%CI)	P-Value	OR(95%CI)	P-Value
Gender						
Male	51(36.43)	081(39.32)				
Female	89(63.57)	125(60.68)	0 .88(0.57-1.38)	0.587		
AGE(years)						
<45	77(55.00)	143(69.42)				
>45	63(45.00)	063(30.58)	1.86(1.19- 2.90)	0.006*	1.24(0 .71- 2.15	0.434
AZT regimen	81(57.86)	72(34.95)	2.56(1.64- 3.97)	0.000*	3.3(1.42- 7.68)	0.005
TDF regimen	47(33.57)	98(47.57)	0.56(0.35-0 .87)	0.010*	2.29(0.97- 5.38)	0.058
D4T regime	12(08.57)	36(17.48)	0.44(0.22- 0.88)	0.021*	-	-
BMI(kg/m^2^)						
<18.5	034( 24.29)	033(16.02)				
>18.5	106(75.71)	173( 83.98)	1.68( 0.98- 2.88)	0.058		
WHO stage						
Stage 1&2	46(32.86)	151(73.30)				
Stage 3&4	94(67.14)	055(26.70)	5.60(3.50- 8.96)	0.000*	5.3(3.15- 8.9)	0.000
Time on ART(MO)	40.4(3-68)	47.2(3-75)	0.99(0.97- 1.0)	0.055		
Baseline CD4						
<200cells/μl	100(71.43)	115(55.83)				
>200cells/μl	040(28.57)	091(44.17)	1.98(1.25- 3.13)	0.004 *	2.27(1.33-3.88)	0.003
Enrolment CD4						
<350cells/μl	121(86.43)	163(79.13)				
>350cells/μl	019(13.57)	043(20.87)	1.70(0.93- 3.03)	0.084		
MCV (fl)						
>100	53(37.86)	023(11.17)				
<100	87(62.14)	183(88.83)	4.85(2.79- 8.42)	0.000*	3.19(1.65- 6.18)	0.001
VL(copies/mm3)						
<10,000	125(89.29)	162(78.64)				
>10,000	015(10.71)	044(21.36)	0.44(0.24- 0.83)	0.011*	0.59(0.28-1.23)	0.164

VL: Viral load, MCV; Mean corpuscular volume, WHO: World Health Organization; OR: Odds Ratio, BMI: Body mass Index

## Discussion

The aim of this study was to determine the magnitude and correlates of moderate to severe anemia in adult HIV positive patients receiving first line HAART at care and treatment centre in northwestern part of Tanzania. In this study the prevalence of moderate to severe anemia was 40.46% and by category of anemia; 30.35%, 26.30% and 14.16% had mild, moderate and severe anemia respectively. Several studies have indicated that moderate to severe anemia is associated with increased disease progression and high mortality in patients receiving ART; with a wide range of prevalence from 11-80.5%. A study from USA (2001) by Moore et al. describing anemia in HIV as being a HB level less than 10mg/dl, reported a prevalence of 11% in patients on HAART [13]. Another study by Lealem et al. from Jima university hospital (Ethiopia) reported a prevalence of moderate to severe anemia in 27.8% with a higher rate of mild anemia occurring in 72.2% of patients who were on HAART [15]; while a study in Nigeria recorded an overall prevalence of anemia in 51.15% of HAART experienced patients and by WHO toxicity grading, grade 2-4(Hb=9.5 to <6.5g/dl) contributed the most of the anemic patients (>49%)[8]. A highest rate of 80.5% was reported by Daka et al from Ethiopia. In this study mild, moderate and severe anemia did occur in 17.7%, 10.4%, and 52.3% of patients respectively [4]. The prevalence of moderate to severe anemia reported by Moore et al. from USA (2002) and Lealem (2013) from Jima are slightly lower than our report, while those reported by Omoregie from Nigeria (2009) and Daka from Ethiopia (2013) are higher than our findings. The differences could be explained by the difference in study design, study subjects and geographical differences. Patients from most of these studies had a shorter time on ART ranging from 6 to 24 months as compared to our study where the median time on ART was 44.5(3-69) months. A study by Daka for example included patients with tuberculosis which he described it as one of strong independent predictor of anemia in HIV patients on HAART [4]. However we generally note that studies from Africa are recording a higher rate of anemia in HIV patients on HAART. This could probably be explained by the fact that, Africa especially the sub Saharan part harbors the highest burden of HIV/AIDS and the associated co morbidities like malnutrition and high prevalence of TB, both of which among other causes could explain the high prevalence of anemia [14]. This is also partly supported by our findings that malnutrition (BMI<18.5) was more common in patients with moderate to severe anemia as compared to those with mild or non anemic counter parts though the difference was not statistically significant (24.29%vs.16.02%, p=0.058)

From previous studies it is suggested that patients with moderate to severe anemia surfer a rapid HIV disease progression and high mortality. In view of this fact the results from this study therefore may have several clinical messages. Of our patients on ART 40.46% are likely to have disease progression and ultimate death. However this high rate of moderate to severe anemia emphasizes on the need to have regular routine screening and early treatment of anemia in patients on HAART. In this study the moderate to severe anemia was strongly predicted by AZT based regimen, advanced clinical stage, low baseline CD4 and macrocytosis. These results are in agreement with several other studies [12, 16]. In this study it was found that patients on AZT regimen were more likely to have moderate to severe anemia as compared to those who were on other regimens other than AZT based (57.86% vs. 34.95, p=0.005). Levine et al. had similar findings, demonstrating a higher rate of anemia (Hb<10g/dl) in AZT regimens than other regimens (41.6% vs.34.3%, P<0.01) [17]. AZT has been shown to inhibit bone marrow activity, reducing blood cells production consequently increasing the risk of developing anemia [18–20]. The rate of Hb gain has been shown to be much slower in AZT regimen thus making AZT containing regimens an independent predictor of persistent anemia [5]. In our study patients with low baseline CD4 count (<200cells/μl) were 2.27 times more likely to have anemia than those with CD4 count of more than 200cell/μl at baseline (OR=2.27, P=0.003). It has also been shown that the likelihood of developing anemia increases with immunological deterioration and the risk of having anemia in patients with CD4<200cells/μl is more than 9 times as compared to patients with CD4>500 cell/μl [15]. Also like in other studies patients in WHO stage 3&4 were also likely to have anemia compared to those in stage 1&2. Both low CD4 counts and advanced WHO stage signify HIV disease progression a state which has been associated with increased risk of anemia [21, 22]. Most patients with severe to moderate anemia were also found to have macrocytosis (MVC>100fl) as compared to those with mild anemia or non anemic counter parts. This suggests that examination of peripheral blood smear in HIV anemic patients on HAART should be taken as one of the important investigations as it gives a hint to the probable cause of the alteration in hematopoiesis. This type of anemia may be caused by medication toxicity including AZT. In this study 153 patients were on AZT based regimen of whom 81(57.86%) had anemia which was mostly macrocytic in nature similar to findings by Jam et al. from Iran where; of the anemic patients on HAART 58% showed features of macrocytosis [22].

## Conclusion

In summary our findings suggest that moderate to severe anemia is very common in HIV patients receiving first line HAART in northwestern Tanzania and since this level of anemia carries high morbidity and mortality patients who are on AZT based regimen and those with advanced HIV parameters should have regular and routine Hb check up to identify anemia at its earliest stage to improve their clinical outcome. **Limitations** This was a cross sectional, single site clinic based study, with a small sample size compared to other studies. The results from this study therefore may not be generalizable. Other causes of anaemia in HIV-infected patients were not exhausted in this study. There was a limitation in the number of tests that could be done to establish the cause of anaemia in this study. Another limitation for this study is that baseline Hb was not tested; no comparison could be made as a result. A longitudinal study is recommended to answer this question.

### What is known about this topic

- In general anemia is a very common non immunological complication of HIV with serious clinical implications. Developed countries have documented a better capacity to deal appropriately with the causes and solution of the most causes of anemia in HIV.
- Most of the diagnostic and treatment measures are still expensive and not readily available for routing care of patients in resource limited countries where the burden of anemia is highest and with complex interaction of its causal factors.

### What this study adds

- This study has demonstrated that even with HAART which is initiated as a standard of care of patients with HIV intended to reverse both the immunological and non immunological complications of HIV, moderate to severe anemia is still a big problem in ART experienced patients especially in resource limited setting like ours. It stresses the importance of screening for anaemia prior to initiation of AZT.
- Additionally this study adds to the existing body of knowledge that identification and correction of causes of anemia may still be complex to handle in resource limited settings. Early HIV diagnosis and timely initiation of ART may potentially reduce the late HIV diagnosis which is one of the risk factors and that at risk patients should strategically be identified and followed up before anemia advances to reduce its serious clinical implications.
